# *TNFA* and *IL10* Polymorphisms and IL-6 and IL-10 Levels Influence Disease Severity in Influenza A(H1N1)pdm09 Virus Infected Patients

**DOI:** 10.3390/genes12121914

**Published:** 2021-11-28

**Authors:** Kalichamy Alagarasu, Himanshu Kaushal, Pooja Shinde, Mahadeo Kakade, Urmila Chaudhary, Vikram Padbidri, Shashikala A. Sangle, Sonali Salvi, Ashish R. Bavdekar, Pradeep D’costa, Manohar Lal Choudhary

**Affiliations:** 1 ICMR-National Institute of Virology, Pune 411001, India; alagarasu@gmail.com (K.A.); hkarya@gmail.com (H.K.); poojashinde799@gmail.com (P.S.); mahadeo_kakade82@yahoo.co.in (M.K.); urmichoudhary1989@gmail.com (U.C.); 2 Jehangir Hospital Research Center, Pune 411001, India; vikram.padbidri@gmail.com; 3 Department of Medicine, BJ Medical College, Pune 411001, India; shashisangle@yahoo.com (S.A.S.); sonalionly@gmail.com (S.S.); 4 KEM Hospital Research Center, Pune 411001, India; ashish.bavdekar1@gmail.com (A.R.B.); pradeepdcosta@yahoo.co.in (P.D.)

**Keywords:** influenza A(H1N1)pdm09, cytokines, gene polymorphisms, TNF-α, interleukins

## Abstract

Cytokines are key modulators of immune response, and dysregulated production of proinflammatory and anti-inflammatory cytokines contributes to the pathogenesis of influenza A(H1N1)pdm09 virus infection. Cytokine production is impacted by single nucleotide polymorphisms (SNPs) in the genes coding for them. In the present study, SNPs in the *IL6*, *TNFA*, *IFNG*, *IL17A*, *IL10,* and *TGFB* were investigated for their association with disease severity and fatality in influenza A(H1N1)pdm09-affected patients with mild disease (*n* = 293) and severe disease (*n* = 86). Among those with severe disease, 41 patients had fatal outcomes. In a subset of the patients, levels of IL-2, IL-4, IL-6, IL-10, TNF, IFN-γ, and IL-17 were assayed in the plasma for their association with severe disease. The frequency of *TNFA* rs1800629 G/A allele was significantly higher in severe cases and survived severe cases group compared to that of those with mild infection (OR with 95% for mild vs. severe cases 2.95 (1.52–5.73); mild vs. survived severe cases 4.02 (1.84–8.82)). *IL10* rs1800896-rs1800872 G-C haplotype was significantly lower (OR with 95% 0.34 (0.12–0.95)), while *IL10* rs1800896-rs1800872 G-A haplotype was significantly higher (OR with 95% 12.11 (2.23–76.96)) in fatal cases group compared to that of the mild group. IL-6 and IL-10 levels were significantly higher in fatal cases compared to that of survived severe cases. IL-6 levels had greater discriminatory power than IL-10 to predict progression to fatal outcome in influenza A(H1N1)pdm09 virus-infected patients. To conclude, the present study reports the association of *TNFA* and IL10 SNPs with severe disease in Influenza A(H1N1)pdm09 virus-infected subjects. Furthermore, IL-6 levels can be a potential biomarker for predicting fatal outcomes in Influenza A(H1N1)pdm09 virus infected subjects.

## 1. Introduction

Pandemic H1N1 influenza (pH1N1), a severe acute respiratory illness (SARI) caused by influenza A(H1N1)pdm09, contributed to increased mortality among those infected with different influenza A subtype viruses [1]. In India, Maharashtra, a state in the western part of the country, accounts for 21% of all the influenza A(H1N1)pdm09 cases and 31% of all pH1N1-related deaths [2]. The progression to SARI depends on multiple factors such as age, obesity, the virulence of the virus, genetics of the host, and host immune response [3]. 

Cytokines, mediators of the immune response, contribute to disease pathogenesis. Influenza A(H1N1)pdm09-infection is known to induce exuberant proinflammatory cytokine response in both respiratory and extra-respiratory tissues [4]. Dysregulated production of tumor necrosis factor (TNF)-α, interleukin (IL)-1β, IL-6, and IL-17A, which contribute to the proinflammatory response, and IL-10, an anti-inflammatory cytokine, were reported in influenza A(H1N1)pdm09 virus infections [5]. Animal studies showed that inhibition of TNF-α averts lethal disease in influenza A(H1N1)pdm09 virus-infected mice [6].

Production of cytokines is affected by the single nucleotide polymorphisms (SNPs) in the genes coding for them. SNPs in the promoter and intron regions are known to affect the stability of the mRNA, transcriptional efficiency, and splicing of the transcribed RNA, and thereby influence cytokine production. Commonly occurring SNPs that are known to influence cytokine levels were reported in the genes coding for TNF-α, IL-6, interferon (IFN)-γ, IL-17A, IL-10, and tumor growth factor (TGF)-β [7,8,9]. SNPs in the cytokine genes were shown to be associated with disease severity in influenza A(H1N1)pdm09 virus infected subjects [10,11,12]. There is a lack of studies investigating the association between cytokine gene polymorphisms and severe influenza A(H1N1)pdm09 infection in India. In the present study, common SNPs in the *IL6* (rs1800795), *TNFA* (rs1800629), *IFNG* (rs2430561), *IL17A* (rs2275913), *IL10* (rs1800896 and rs1800872), and *TGFB* (rs1800470 and rs1800471) were investigated for their association with severe disease and fatality in influenza A(H1N1)pdm09 virus infected patients. In a subset of the patients, levels of IL-2, IL-4, IL-6, IL-10, IL-17, TNF-α, and IFN-γ were assayed in the plasma for their association with severe disease. The effect of SNPs on the plasma level of the cytokines was also studied.

## 2. Materials and Methods

### 2.1. Study Subjects

Patients with a history of laboratory confirmed influenza A(H1N1)pdm09 virus infection or ongoing treatment were contacted for enrolment in the study, and blood samples were collected from the consenting study subjects. Patients with influenza A(H1N1)pdm09 virus infection admitted to the collaborating hospitals were also included in the study. All the patients included in the study were affected with influenza A(H1N1)pdm09 virus infection during 2013 to 2018. Written informed consent was obtained from all the participants or their legal guardians before sample collection. The study was reviewed and approved by the institutional ethics committee of ICMR-National Institute of Virology and the collaborating hospitals. Influenza A(H1N1)pdm09 virus infection in the study subjects during the acute phase of the disease was confirmed by a real-time RT-PCR assay. DNA was extracted from the blood samples using a commercial DNA extraction kit (QIAamp blood DNA midi kit, Qiagen, Hilden, Germany) according to the protocol given by the manufacturer. Case history sheets filled by the treating physicians were used to classify the patients into those with mild disease and severe disease. Patients admitted to intensive care unit and requiring ventilator support or the patients with fatal outcomes were classified as those with severe influenza A(H1N1)pdm09 virus disease [10,13]. All the other patients were considered as those with mild disease. All the patients were treated according to the clinical management protocols provided by the directorate general of health services, ministry of health and family welfare, Government of India (https://main.mohfw.gov.in/sites/default/files/2366426352.pdf, accessed on 19 November 2021).

### 2.2. Genotyping of SNPs in the Cytokine Genes

SNPs in the *IL6* (rs1800795), and *IFNG* (rs2430561) genes were assayed using restriction fragment length polymorphism-based method as reported earlier [14,15]. *IL17A* rs2275913 was studied using the TaqMan SNP genotyping method according to the manufacturer’s instructions. SNPs in the *TNFA* (rs1800629), *IL10* (rs1800896 and rs1800872), and *TGFB* (rs1800470 and rs1800471) genes were investigated using polymerase chain reaction with allele specific primers, as described previously [16,17,18].

### 2.3. Investigation of Cytokine Levels in the Plasma of Influenza A(H1N1)pdm09 Virus Infected Cases

The cytokine levels were investigated in a subset of samples. The plasma samples collected during the acute phase (within a week of disease onset) of influenza A(H1N1)pdm09 virus infection from fatal (*n* = 28) and survived cases (*n* = 28) were examined for the cytokine profile. Plasma samples collected from patients who recovered from mild A(H1N1)pdm09 infection (*n* = 21) and healthy controls (*n* = 11) were also assessed for cytokine levels. The level of cytokines IL-2, IL-4, IL-6, IL-10, TNF, IFN-γ, and IL-17A were analyzed using BD™ Cytometric Bead Array Human Th1/Th2/Th17 Cytokine Kit (BD Biosciences, San Jose, CA, USA), as per manufacturer’s instructions. Briefly, 50 µL plasma was treated with capture beads and phycoerythrin (PE) conjugated detection antibody. The standards provided by the manufacturer were used to prepare the standard curve for each cytokine. Then, the samples, as well as the standards, were acquired and analyzed on a BD FACSCalibur^TM^ flow cytometer (BD Biosciences, San Jose, CA, USA) using BD CellQuestTM Pro software (v5.1). The fcs files were analyzed using flow cytometric analysis program array^TM^ software (v3.0). The detection sensitivity of IL-2, IL-4, IL-6, IL-10, IFN-γ, TNF-α, and IL-17A were 2.6, 4.9, 2.4, 4.5, 3.8, 3.7, and 18.9 pg/mL, respectively.

### 2.4. Statistical Analysis

For genetic association studies, statistical comparisons were carried out using an online SNP stats program [19] or SPSS version 17 as described earlier [20]. The strength of association was represented in terms of odds ratio (OR) with 95% confidence intervals (CI). The OR with 95% CI was adjusted for age and gender. For cytokine levels and their association with genotypes or disease severity, the Mann–Whitney U test was used to compare two groups while the Kruskal–Wallis test with multiple corrections was used to compare multiple groups. Receiver operating curve analysis was performed to assess the utility of cytokines as biomarkers in predicting fatal outcomes. Graphpad prism software version 7 was used for the analysis.

## 3. Results

### 3.1. Demographic Characteristics and Disease Profile of the Study Subjects

A total number of 379 influenza A(H1N1)pdm09 virus-infected patients from Pune and its adjoining districts, Maharashtra, western India were included in the study. Among the patients, 86 were categorized as severe cases based on the requirement of ventilator support and/or fatal outcome. Among the severe cases, 41 patients had fatal outcomes, while the remaining 45 survived. The remaining 293 cases were categorized as having a mild disease. The ratio of males to females is 1.15 for mild cases and 0.97 for severe cases. Severe cases had significantly higher age as compared to mild cases (Table 1). Within the severe cases group, the ratio of male to female was 1.03 among survived severe cases and 1.27 in the fatal cases group. The mean age ± standard deviation (sd) was 51 ± 14.6 in the fatal cases group while it was 50 ± 12.6 in the survived severe cases group and the difference was not statistically significant.

### 3.2. Allele, Genotype, and Haplotype Frequencies in Influenza A(H1N1)pdm09 Virus-Infected Cases

Comparison of allele frequencies among the study groups revealed that the frequency of *TNFA* rs1800629 A allele was significantly higher in severe cases and survived severe cases group compared to that of those with mild infection (OR with 95% for mild vs. severe cases 2.72 (1.47–4.99); mild vs. survived severe cases 3.49 (1.68–6.99)). Though the frequency of *TNFA* rs1800629 A allele was higher in fatal cases compared to that of mild cases, it was not statistically significant. The allele frequencies of other SNPs were not different between the influenza A(H1N1)pdm09 infected patient groups (Table 2).

Comparison of genotype frequencies of different SNPs among the study groups revealed that the frequency of *TNFA* rs1800629 G/A allele was significantly higher in severe cases and survived severe cases group compared to that of those with mild infection (OR with 95% for mild vs. severe cases 2.95 (1.52–5.73); mild vs. survived severe cases 4.02 (1.84–8.82)). Though the frequency of *TNFA* rs1800629 G/A allele was higher in fatal cases compared to mild cases, it was not statistically significant. The genotype frequencies of other SNPs were comparable between the influenza A(H1N1)pdm09-infected patient groups (Table 3).

Comparison of IL10 haplotype frequencies revealed that *IL10* rs1800896–rs1800872 G-C haplotype was significantly lower while *IL10* rs1800896–rs1800872 G-A haplotype was significantly higher in fatal cases group compared to mild cases (Table 4). Haplotype frequencies of TGFB SNPs were not different between different categories of influenza A (H1N1)pdm09 virus-infected patient groups.

### 3.3. Elevated Plasma IL-6 and IL-10 Level among Fatal Cases during the Acute Phase of Influenza A(H1N1)pdm09 Virus Infection

**Figure 1:**
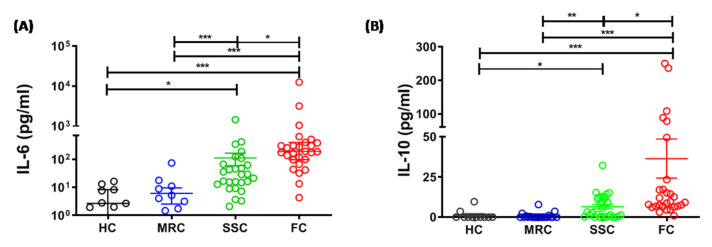
(**A**). IL-6 levels in healthy controls (HC), mild recovered cases (MRC), survived severe cases (SSC), and fatal cases (FC). (**B**). IL-10 levels in HC, MRC, SSC, and FC. Circles represent the cytokine level of individual subjects and lines indicate median and interquartile range. * *p* < 0.05; ** *p* < 0.01; *** *p* < 0.001.

The proinflammatory cytokine, IL-6 was found to be significantly higher during the acute phase of fatal cases (Mean ± SE, 812 pg/mL ± 451.3, *p* <0.001) and survived severe cases (Mean ± SE, 112.6 pg/mL ± 53, *p* < 0.05) compared to that of healthy controls (Mean ± SE, 5.039 pg/mL ± 1.69) and mild recovered cases (Mean ± SE, 6.11 pg/mL ± 3.57). Also, IL-6 level was found significantly higher (*p* < 0.05) in fatal cases compared to that of survived severe cases (Figure 1A).The plasma IL-10 level was found significantly higher during the acute phase of fatal cases (Mean ± SE, 36.44 pg/mL ± 12.18, *p* < 0.001) and survived severe cases (Mean ± SE, 6.567 pg/mL ± 1.43, *p* < 0.05) compared to that of healthy controls (Mean ± SE, 1.20 pg/mL ± 0.90) and mild recovered cases (Mean ± SE, 0.76 pg/mL ± 0.41). IL-10 level was significantly higher in fatal cases compared to that of survived severe cases (*p* < 0.05) (Figure 1B). The other evaluated cytokines such as IL-2, IL-4, IFN-γ, TNF-α, and IL-17A, were observed only in few patients, and the levels were comparable between the study groups.

### 3.4. IL-6 and IL-10 Levels among Influenza A(H1N1)pdm09 Virus-Infected Subjects with Different Genotypes of IL-6 and IL-10

**Figure 2:**
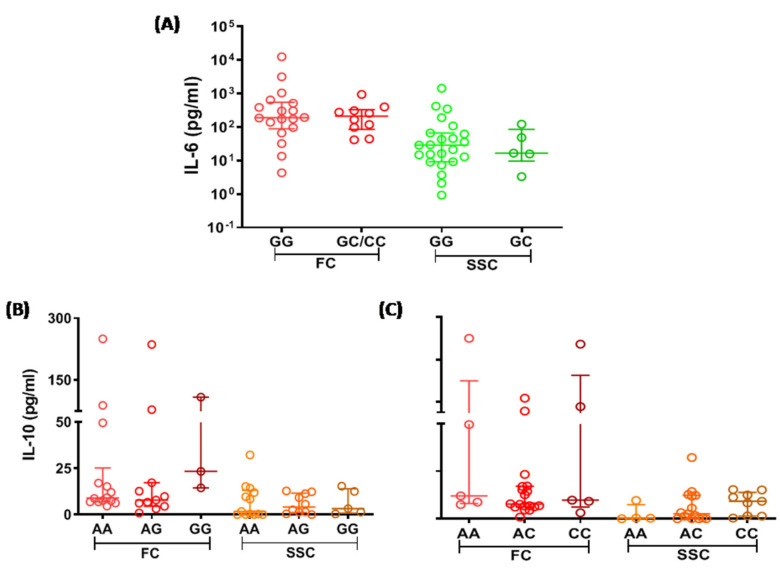
(**A**) IL-6 levels in survived severe cases (SSC) and fatal cases (FC) with different *IL6* rsrs1800795 genotypes. C/C and G/C genotypes were clubbed together due to fewer subjects with C/C genotype. Circles represent cytokine level of individual subjects and lines indicate median and interquartile range. (**B**) IL-10 levels in survived severe cases (SSC) and fatal cases (FC) with different *IL10* rs1800896 genotypes. (**C**) IL-10 levels in survived severe cases (SSC) and fatal cases (FC) with different *IL10* rs1800872 genotypes.

When IL-6 and IL-10 levels were compared between levels among influenza A(H1N1)pdm09 virus-infected subjects with different genotypes of IL-6 and IL-10, the cytokine levels were not significantly different between subjects with different *IL6* or *IL10* genotypes. However, irrespective of the *IL6* and *IL10* genotypes, the cytokine levels were elevated in fatal cases compared to that of survived severe cases (Figure 2A–C).

### 3.5. Utility of IL-6 and IL-10 Levels as A Marker for Predicting Fatality in Influenza A(H1N1)pdm09 Virus-Infected Subjects

**Figure 3:**
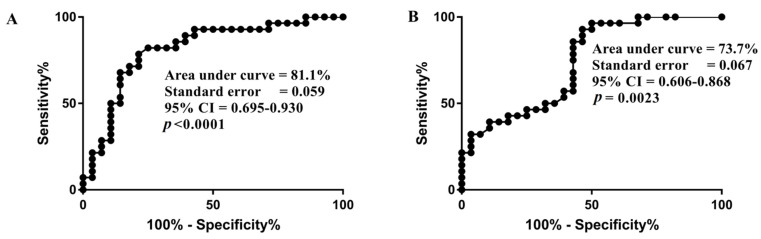
Receiver operating curve based on (**A**) IL-6 and (**B**) IL-10 levels in survived severe cases and fatal cases.

The utility of IL-6 and IL-10 levels in predicting fatal outcomes in influenza A(H1N1)pdm09 virus infected subjects with severe disease was investigated using receiver operating curve analysis. The analysis revealed that the area under curve (AUC) value for IL-6 in discriminating survived severe cases and fatal cases was 0.81 (*p* < 0.0001) while it was 0.73 (*p* < 0.01)for IL-10 (Figure 3A,B).

## 4. Discussion

Cytokines are the key mediators of inflammation and were associated with the disease severity in influenza A(H1N1)pdm09 virus infection [21,22,23]. In the present study, the SNPs in the *IL6*, *TNFA*, *IFNG*, *IL17A*, *IL10,* and *TGFB* genes were investigated for their association with disease severity and fatality in influenza A(H1N1)pdm09 virus-infected subjects. The results revealed that *TNFA*rs1800629 A allele and G/A genotype were associated with severe disease but not fatal outcomes. Our earlier study [15] with a smaller sample size also reported the association of *TNFA*rs1800629 A allele and G/A genotype with severe disease, and this was also confirmed in the present study. Contradicting our study, a study of a Mexican population showed the association of *TNFA*rs1800629G allele with severe disease [24]. *TNFA* rs1800629 is located in the promoter region, and this SNP, in combination with other SNPs, might influence TNF-α production. *TNFA* rs361525 was shown to be associated with severe disease in Mexican population [10]. Thus, the haplotype structure might vary between populations and influence genetic associations of TNFA SNPs with the disease in different populations. The lack of association of *TNFA*rs1800629 with fatality suggests the role of additional factors including other genetic factors and comorbidities in progression to a fatal outcome and needs investigation. 

The results also suggested that*IL10* rs1800896-rs1800872 G-C haplotype was associated with protection to fatal outcome, while *IL10* rs1800896-rs1800872 G-A haplotype was associated with fatal outcomes in influenza A(H1N1)pdm09 virus infected-subjects. Similar to our study, *IL10* rs1800896-rs1800872 G-A haplotype was reported to be associated with protection against severe disease in a Mexican population [24]. *IL10* rs1800896-rs1800872 G-A haplotype might be associated with dysregulated production of IL-10 while *IL10* rs1800896-rs1800872 G-C haplotype might be associated with optimal IL-10 production. Thus, these *IL10* haplotypes might be associated with contrasting outcomes. The present study also reported the lack of association between SNPs in the *IL6*, *IFNG*, *IL17A,* and *TGFB* genes and disease severity of Influenza A(H1N1)pdm09 virus infection.

The present study demonstrated an elevated level of IL-6, indicative of raised inflammatory responses during the acute phase of influenza A(H1N1)pdm09 virus infection. Previous studies also indicated a higher level of IL-6 in severe influenza A(H1N1)pdm09 disease [21,22,25]. Besides, our study also revealed an increased level of plasma IL-10 during the acute phase of the infection and is in line with the previous findings [23,26]. Together, our study reported that there is a surge in plasma IL-6 and IL-10 level during the acute phase of influenza A(H1N1)pdm09 virus infection and also, this cytokine surge is more pronounced in fatal cases, as compared to that of severe survived cases, indicating their critical role in the pathogenesis of severe influenza A(H1N1)pdm09 disease. In some instances, IL-6 may get altered to resemble IL-10, a potent inhibitor of dendritic cells and macrophages [27,28], and thereby suppressing their microbicidal activities and production of proinflammatory cytokines. Thus, the immunosuppressive effect of IL-10 in synergy with IL-6, probably supports viral persistence or secondary infection, resulting in the more severe clinical outcome in influenza A(H1N1)pdm09 virus [23]. The utility of IL-6 and IL-10 in discriminating fatal and nonfatal cases was also investigated. The results revealed that IL-6 has greater discriminatory power than IL-10 and can be utilized as a biomarker to predict fatal outcomes in influenza A(H1N1)pdm09 virus-infected subjects and undertake better treatment strategies to prevent the same. Since IL-6 is nonspecifically upregulated in all type of infections [29], it cannot be used as a biomarker of influenza A(H1N1)pdm09 virus infection but can be used as a biomarker to predict fatal outcomes in subjects with confirmed influenza A(H1N1)pdm09 virus infection.

The study also revealed that investigated *IL6* and *IL10* SNPs were not associated with plasma IL-6 and IL-10 levels. An earlier study from the Mexican population also reported no association between plasma cytokine levels and cytokine gene SNPs [24]. However, a study using respiratory syncytial virus stimulated macrophages showed the association between cytokine levels and cytokine SNPs [8]. It is plausible that association between plasma cytokine levels and cytokine SNPs are not usually observed since plasma cytokines represent total cytokines produced by different cell types under specific stimulation as well as nonspecific production of cytokines.

It was shown that age, obesity, underlying other chronic diseases, secondary bacterial coinfections, and early administration of oseltamivir influence disease severity in influenza A(H1N1)pdm09 virus-infected subjects [30]. In the present study, details of the existing comorbidities in the study subjects were not available and are a limitation. 

## 5. Conclusions

The present study suggests that *TNFA* and *IL10* SNPs and IL-6 and IL-10 levels are associated with disease severity in influenza A(H1N1)pdm09 virus-infected subjects. Further, IL-6 levels might have utility as a biomarker for predicting fatal outcomes in influenza A(H1N1)pdm09 virus-infected subjects.

## Figures and Tables

**Table 1:** Demographic characteristics of influenza A(H1N1)pdm09-infected patients with different grades of disease severity.

Demographic Characteristics	Mild Cases (*n* = 293)	Severe Cases (*n* = 86)	*p*-Value
Age [mean years ± standard deviation] Males [n (%)] Females [n (%)]	39.1 ± 18.0 144 (49.1%) 149 (50.8%)	50.0 ± 13.0 46 (53.5%) 40 (46.5%)	<1 × 10^−6^ − 0.6447

**Table 2:** Percent allele frequencies of cytokine gene polymorphisms in different Influenza A(H1N1)pdm09 patient groups.

SNP	Alleles	Percent Minor Allele Frequency *n*(%) ^A^	Mild Cases vs. Severe Cases/Severe Survived Cases/Fatal Cases	
			Odds Ratio (95% CI)	*p*-Value
*IL6* rs1800795 Mild cases Severe cases Survived severe cases Fatal cases *TNFA* rs1800629 Mild cases Severe cases Survived severe cases Fatal cases *INFG*rs2430561 Mild cases Severe cases Survived severe cases Fatal cases *IL17A* rs2275913 Mild cases Severe cases Survived severe cases Fatal cases *IL10* rs1800896 Mild cases Severe cases Severe survived cases Fatal cases *IL10* rs1800872 Mild cases Severe cases Severe survived cases Fatal cases *TGFB* rs1800470 Mild cases Severe cases Severe survived cases Fatal cases *TGFB*rs1800471 Mild cases Severe cases Severe survived cases Fatal cases	G>C G>A A>T G>A G>A C>A T>C G>C	75 (12.8) 22 (12.8) 10 (11.1) 12 (14.6) 27 (4.6) 20 (13.1) 13 (14.4) 7 (8.2) 218 (37.2) 61 (35.4) 25 (27.8) 36 (43.9) 220 (39.1) 58 (34.1) 31 (35.2) 27 (32.9) 150 (25.6) 40 (23.2) 23 (25.5) 17 (20.7) 252 (43.0) 80 ( 46.5) 42 (46.7) 38 (46.3) 278 (47.6) 80 (47.0) 43 (47.8) 37 (46.2) 45 (7.7) 16 (9.7) 8 (8.9) 8 (9.7)	− 0.99 (0.59–1.64) 0.85 (0.40–1.66) 1.16 (0.58–2.20) − 2.72 (1.47–4.99) 3.49 (1.68–6.99) 1.93 (0.75–4.45) − 0.93 (0.65–1.32) 0.65 (0.39–1.05) 1.32 (0.82–2.11) − 0.85 (0.59–1.22) 0.89 (0.55–1.42) 0.81 (0.49–1.31) − 0.88 (0.59–1.31) 0.99 (0.59–1.65) 0.76 (0.42–1.32) − 1.15 (0.82–1.62) 1.16 (0.74–1.81) 1.14 (0.72–1.82) − 0.98 (0.69–1.38) 1.01 (0.64–1.57) 0.95 (0.59–1.51) − 1.23 (0.66–2.22) 1.17 (0.50–2.50) 1.30 (0.55–2.78)	− 0.999 0.668 0.639 − 0.002 0.001 0.155 − 0.682 0.081 0.246 − 0.384 0.649 0.397 − 0.539 0.994 0.346 − 0.416 0.515 0.569 − 0.901 0.974 0.823 − 0.489 0.672 0.508

A: numbers represent allelic count. Numbers in parenthesis represent percentage.

**Table 3:** Percent genotype frequencies of cytokine gene polymorphisms in Influenza A(H1N1)pdm09 patient groups.

SNP and Genotypes	Genotypic Count (%) in				Mild vs. Severe Cases	Mild vs. Severe Survived Cases	Mild vs. Fatal Cases
	Mild Cases	Severe Cases	Survived Severe Cases	Fatal Cases	Odds Ratio with 95% Confidence Intervals		
*IL6* rs1800795 G/G G/C C/C *TNFA* rs1800629 G/G G/A *INFG*rs2430561 A/A A/T T/T *IL17A* rs2275913 G/G G/A A/A *IL10* rs1800896 A/A A/G G/G *IL10* rs1800872 C/C C/A A/A *TGFB* rs1800470 T/T T/C C/C *TGFB*rs1800471 G/G G/C	224 (76.7) 61 (20.9) 7 (2.4) 266 (90.8) 27 (9.2) 114 (38.9) 140 (47.8) 39 (13.3) 115 (39.5) 132 (45.4) 44 (15.1) 168 (57.3) 100 (34.1) 25 (8.4) 101 (34.5) 132 (45.0) 60 (20.0) 87 (29.8) 132 (45.2) 73 (25.0) 248 (84.6) 45 (15.4)	65 (75.6) 20 (23.3) 1 (1.2) 66 (76.7) 20 (23.3) 34 (39.5) 43 (50.0) 9 (10.5) 40 (47.1) 32 (37.6) 13 (15.3) 55 (64.0) 22 (25.6) 9 (10.5) 22 (25.6) 48 (55.8) 16 (18.6) 25 (29.4) 40 (47.1) 20 (23.5) 70 (81.4) 16 (18.6)	35 (77.8) 10 (22.2) 0 32 (71.1) 13 (28.9) 23 (51.1) 19 (42.2) 3 (6.7) 20 (45.5) 17 (38.6) 7 (15.9) 28 (62.2) 11 (24.4) 6 (13.3) 12 (26.7) 24 (53.3) 9 (20.0) 13 (28.9) 21 (46.7) 11 (24.4) 37 (82.2) 8 (17.8)	30 (73.2) 10 (24.4) 1 (2.4) 34 (82.9) 7 (17.1) 11 (26.8) 24 (58.5) 6 (14.6) 20 (48.8) 15 (36.6) 6 (14.6) 27 (65.8) 11 (26.8) 3 (7.3) 10 (24.4) 24 (58.5) 7 (17.1) 12 (30.0) 19 (47.5) 9 (22.5) 33 (80.5) 8 (19.5)	1.00 1.14 (0.63–2.07) 0.80 (0.10–6.77) 1.00 2.95 (1.52–5.73) * 1.00 1.08 (0.63–1.84) 0.94 (0.40–2.19) 1.00 0.72 (0.42–1.24) 0.87 (0.41–1.85) 1.00 0.63 (0.35–1.1) 0.94 (0.40–2.21) 1.00 1.77 (0.98–3.21) 1.50 (0.71–3.18) 1.00 1.06 (0.59–1.91) 0.86 (0.43–1.73) 1.00 1.21 (0.63–2.32)	1.00 1.02 (0.47–2.21) 0.00 1.00 4.02 (1.84–8.82) ^@^ 1.00 0.67 (0.34–1.31) 0.46 (0.13–1.66) 1.00 0.81 (0.40–1.65) 0.97 (0.37–2.50) 1.00 0.62 (0.29–1.32) 1.29 (0.47–3.54) 1.00 1.61 (0.75–3.45) 1.50 (0.58–3.88) 1.00 1.06 (0.50–2.28) 0.97 (0.40–2.35) 1.00 1.15 (0.49–2.68)	1.00 1.28 (0.58–2.81) 1.76 (0.20–15.31) 1.00 2.04 (0.80–5.17) 1.00 1.85 (0.85–4.03) 2.04 (0.68–6.10) 1.00 0.67 (0.32–1.39) 0.79 (0.29–2.18) 1.00 0.61 (0.28–1.33) 0.60 (0.16–2.21) 1.00 1.95 (0.87–4.36) 1.47 (0.51–4.18) 1.00 1.07 (0.49–2.37) 0.79 (0.31–2.02) 1.00 1.30 (0.55–3.09)

* *p* = 0.0018; ^@^ *p* = 9 × 10^−4^.

**Table 4:** Percent haplotype frequencies of *IL10* and *TGFB* polymorphisms in influenza A(H1N1)pdm09 patient groups.

Haplotypes	Percent Predicted Haplotype				Mild vs. Severe Cases	Mild vs. Severe Survived Cases	Mild vs. Fatal Cases
	Mild Cases	Severe Cases	Severe Survived Cases	Fatal Cases	Odds Ratio with 95% Confidence Intervals		
*IL10* rs1800896-rs1800872 A-A A-C G-C G-A *TGFB* rs1800470-rs1800471 T-G C-G C-C T-C	41.9 32.5 24.5 1.1 50.8 41.5 6.1 1.6	43.2 33.6 19.9 3.3 49.9 40.8 6.2 3.1	45.3 29.2 24.2 1.4 48.0 43.1 4.7 4.2	39.4 39.9 13.8 7.0 51.8 38.4 7.7 2.00	1.00 0.92 (0.60–1.40) 0.66 (0.41–1.07) 3.21 (0.75–13.69) 1.00 0.98 (0.68–1.40) 0.87 (0.36–2.12) 2.84 (0.60–13.50)	1.00 0.77 (0.44–1.34) 0.83 (0.48–1.45) 1.19 (0.12–11.98) 1.00 1.08 (0.68–1.71) 0.68 (0.17–2.65) 3.91 (0.67–22.86)	1.00 1.25 (0.69–2.25) 0.34 (0.12–0.95) * 12.11 (2.23–76.96) ^@^ 1.00 0.88 (0.55–1.43) 1.12 (0.39–3.24) 1.74 (0.17–18.18)

* *p* = 0.041; ^@^ *p* = 0.005.

## Data Availability

The data presented in this study are available in the article.
